# Relationship between workplace social capital and suicidal ideation in the past year among employees in Japan: a cross-sectional study

**DOI:** 10.1186/s12889-019-7244-9

**Published:** 2019-07-09

**Authors:** Daisuke Hori, Soshi Takao, Ichiro Kawachi, Yuh Ohtaki, Christina-Sylvia Andrea, Tsukasa Takahashi, Nagisa Shiraki, Tomohiko Ikeda, Yu Ikeda, Shotaro Doki, Yuichi Oi, Shinichiro Sasahara, Ichiyo Matsuzaki

**Affiliations:** 1 Faculty of Medicine, University of Tsukuba, 1-1-1 Tennodai, Tsukuba, 305-8575 Japan; 2 Department of Epidemiology, Graduate School of Medicine, Dentistry and Pharmaceutical Sciences, Okayama University, Okayama, Japan; 3 Department of Social and Behavioral Sciences, Harvard T.H.Chan School of Public Health, Boston, USA; 4 Hospital Bando, Bando, Japan; 5 Graduate School of Comprehensive Human Sciences, University of Tsukuba, Tsukuba, Japan; 6 International Institute for Integrative Sleep Medicine, Tsukuba, Japan

**Keywords:** Workplace social capital, Suicidal ideation, Researcher, Tsukuba Science City, Japan

## Abstract

**Background:**

A growing body of evidence has demonstrated the associations between social capital and health. In residential or geographical areas, social capital has attracted attention for its protective effects against suicide. However, to this date, the relationship between social capital and suicidal ideation is not fully elaborated in the occupational setting. Therefore, the aim of the present study was to examine the association between workplace social capital and suicidal ideation in the past year among employees in Japan.

**Methods:**

A cross-sectional, web-based survey was conducted in February/March 2017 via an anonymous self-administered questionnaire distributed to workers in Tsukuba Science City, Japan. Binomial logistic regressions were used to calculate the odds ratios (ORs) and 95% confidence intervals (CIs) for suicidal ideation in the past year, controlling for age group, marital status, educational attainment, and annual household income. The results were shown stratified by sex and occupation.

**Results:**

In total, 7255 of 19,481 workers responded, out of which we could analyze 6325 responses (4030 men, 2295 women). The prevalence of suicidal ideation in the past year was 5.9% for men and 7.8% for women. Low workplace social capital was statistically significantly associated with suicidal ideation both for men (OR = 2.57, 95% CI = 1.72–3.83) and for women (OR = 1.75, 95% CI = 1.15–2.66), compared with high workplace social capital after controlling for socioeconomic factors.

**Conclusion:**

Higher workplace social capital was associated with a reduced risk of suicidal ideation in the past year. Promoting workplace social capital could contribute to preventing suicide among employees in Japan.

## Background

Every year, it is estimated that more than 800,000 people around the world commit suicide [1], and among all countries, Japan has the ninth highest suicide rate. Starting in 1998, accompanying an economic crisis and recession, the annual number of deaths due to suicide in Japan exceeded 30,000, a trend that would continue for 14 consecutive years [2]. Although this number decreased after 2009, more than 21,000 people still died from suicide in Japan in 2017, representing a crude suicide rate of 16.8 per 100,000 (24.0 for men; 10.0 for women) [3]. Among the suicide victims in Japan in 2017, 37.0% were workers.

Japan is infamous for *karo-shi*, a term which refers to a worker’s sudden death as a result of overwork or work-related exhaustion [4]. *Karo-shi* is administratively defined as a worker’s sudden death from cerebrovascular or cardiovascular disease or suicide because of mental illness (e.g., depression). In 2014, an act promoting measures for *karo-shi* came into effect. Furthermore, in 2015, the Japanese government started a stress check program focusing on the primary prevention of mental health problems among workers [5]. Nevertheless, *karo-shi* received more attention and caused more controversy in 2015 as a result of the suicide of a young woman aged 24 after she worked more than 100 h of overtime in a month at an advertising company [6]. Suicide among workers leads to marked social and economic losses and remains a serious occupational and public health concern.

Inequalities in the incidence of suicide in Japan by occupation and industry have been reported. A steep increase in suicide risk for male specialists and technical workers, who had the lowest suicide risk in 1975, occurred in 2005 [7]. Among men, those working in the scientific research and professional and technical services industries had the highest incidence rates for overwork-related suicide between 2005 and 2010 [8]. Researchers usually have demanding workloads and face long working hours. It is plausible that researchers and professional and technical workers lead more stressful lives because of the rapid advances over the past several decades in science, technology, and information media. However, research on the determinants of suicidal ideation among researchers and professional workers remains scarce.

Although suicide is a serious problem, it is also a preventable health outcome. A national survey found that about one-fourth of Japanese people have seriously contemplated suicide [9]. Suicidal ideation is a risk factor associated with attempted or completed suicide [10]. Therefore, possible countermeasures against suicide involving the alleviation of suicidal ideation need to be explored. A systematic review suggested that workplace suicide prevention initiatives have beneficial effects [11]. The common contents of suicide prevention programs in the workplace focus on education and training for individuals and managers, as well as on reinforcing social relationships, leveraging both internal and external resources [12]. A sense of isolation within a group has been shown to lead to an increased risk for suicidal behaviors [1]. Therefore, the present study highlights the role of social ties, namely workplace social capital, in suicide prevention. Social capital is defined as the resources such as interpersonal trust and norms of reciprocity that are available within social groups [13].

In residential or geographical areas, social capital has attracted attention for its protective effects against suicide. A growing number of studies have investigated the relationship between social capital and suicide-related factors [14–23]. These studies demonstrated empirical support for a positive association between high social capital and a low suicide rate, less suicidal behavior, and decreased suicidal ideation. Social capital accrues wherever people spend much of their time [24]. Therefore, in addition to social capital in neighborhoods, an increasing body of evidence has also demonstrated that workplace social capital is beneficial for employees across a range of health outcomes. For example, workplace social capital has been shown to be inversely associated with smoking status [25, 26], burnout [27], and mortality rates [28].

It is plausible that workplace social capital could be an essential resource to help prevent suicide among employees, similar to neighborhood social capital for residents. Although the underlying mechanism connecting workplace social capital to health achievements remains controversial, workplace social capital is believed to increase the likelihood of access to various types of social resources in times of need. For example, workers with high workplace social capital may find it easier to get help from colleagues or supervisors and to gain access to mental health care when facing stressful events. Another explanation is that in a workplace with group health consciousness norms, such as a company policy to promote smoking cessation, workplace social capital serves to reinforce healthy behaviors.

To date, there have been 11 studies examining the association between workplace social capital and mental health. Among them, 6 studies assessed psychological distress as a main outcome measured by K6 [29–31], GHQ (Genral Health Questionnaire)-12 [32, 33], and GHQ-28 [34]. Other 2 studies assessed well-being measured by WHO-five well-being index [35, 36]. The remaining 3 studies assessed depression as a main outcome measured by web-based survey based on WHO Composite International Diagnostic Interview (WHO-CIDI) [37], prescription of antidepressant and self-reported physician-diagnosed depression [38, 39]. Although suicidal ideation was asked by 2 studies using GHQ-28 or WHO-CIDI, the sample sizes were limited (*n* = 340 or *n* = 929). Prescription of antidepressant and self-reported physician-diagnosed depression suggest the existence of suicidal ideation only indirectly. Therefore, we considered an impact of workplace social capital on suicidal ideation.

Therefore, we conducted a cross-sectional, web-based survey focusing on suicidal ideation among workers in Tsukuba Science City. Tsukuba Science City is one of the largest research park cities in the world. Many researchers at Tsukuba Science City are involved in work related to advanced sciences and technology. The Japanese government established this science park city in a countryside plain in the 1970s, and practically forced researchers and professionals to move to the area. Problems related to their mental health were once sensationally reported by mass media in 1980s, because there was a rumor that the suicide mortality in Tsukuba Science City was high, although this was later found to be untrue [40].

We hypothesized that workplace social capital is protective against suicide. As no previous study has fully elucidated the relationship between workplace social capital and suicidal ideation among workers, the purpose of the present study was to investigate the association between individual levels of workplace social capital among researchers and professional workers and suicidal ideation in the past year.

## Methods

### Study design and participants

Tsukuba Science City is located northeast of Tokyo in Ibaraki Prefecture, Tsukuba Science City, which covers an area of about 27 km^2^, features two universities and a number of public and private research institutes with a population of about 230,000, including more than 20,000 researchers. As of 2017, the Tsukuba Science City Network (TSCN) consisted of 89 organizations around the city, including research institutions, universities, educational foundations, local governments, and private companies. Every 5 years since 1986, TSCN has conducted the Living Condition and Workplace Stress Survey, which aims to investigate the mental health status, living conditions, and workplace environments of workers in Tsukuba [41, 42]. The current 7th survey was conducted on workers who were members of the TSCN in February/March 2017. A total of 53 organizations, mainly comprising research and educational institutions, with a total of 19,481 workers agreed to participate.

The workers were contacted via e-mail through the general affairs department of each institution. A URL described in the e-mail linked to a self-administered questionnaire form. At the outset, participants were able to choose either the Japanese or the English version of the questionnaire. The questionnaire items in the survey involved suicidal ideation, workplace social capital, age group, marital status, educational attainment, annual household income (Japanese yen), and occupation. All participants remained anonymous, so no data regarding to which institute the participants belonged were obtained.

The questions regarding suicidal ideation were asked in two steps. First, the respondents were asked “Have you ever seriously thought of committing suicide?”, with the answering options of “yes”, “no”, and “non-response”. Respondents who answered “yes” proceed to the next question, “Have you thought of committing suicide in the past year?”, with the same answering options as the first one. We defined respondents who answered “Yes” to both of the first and second questions as a group of having suicidal ideation in the past year. We excluded the respondents from the analyses, who answered “non-response” to either the first or the second question. The remaining respondents were defined as a group without suicidal ideation in the past year. These questions were derived from a survey on attitudes toward suicide conducted by the Japanese Ministry of Health, Labour and Welfare [9].

Workplace social capital was assessed using a validated and psychometrically-tested eight-item measure, developed by Kouvonen et al. [43]. Odagiri et al. [44] confirmed acceptable internal consistency, reliability, and validity for the Japanese version of the questionnaire. Responses were given on a five-point rating scale (from 1 = “totally disagree” to 5 = “totally agree”, apart from one item for which the responses were from 1 = “very little” to 5 = “very much”). Workplace social capital has two dimensions: cognitive and structural. The cognitive dimension consists of the trust, solidarity, and reciprocity shared among members of the same group, while the structural dimension consists of transparent decision-making processes, accountable leaders, collective action, and mutual responsibility. A higher score indicates a higher level of social capital. The mean scores were calculated for these eight items (range = 1.00–5.00). To date, workplace social capital has no standard cutoff point; therefore, it was divided into the following four categories based on quartile distributions [43]: low (≤ 3.125), mid-low (> 3.125, ≤ 3.75), mid-high (> 3.75, ≤ 4.00), and high (> 4.00). Cronbach’s alphas were 0.940 for men and 0.935 for women.

Occupation was derived from the item: “Which of the following best describes your job?”, with the answer options as “researcher/academic”, “technician/engineer”, and “clerk/administration”. Age group was categorized into the following four groups: “20–29”, “30–39”, “40–49”, and “50–65 years”. Marital status was divided into three groups: “married”, “single”, and “divorced/widowed”. Educational attainment was categorized into four groups: “high school”, “college, national institute of technology, or vocational school”, “university”, and “graduate school”. Annual household income (Japanese yen) was classified into four groups: < 4 million, 4–8 million, 8–12 million, and ≥ 12 million.

### Statistical analysis

Since workplace social capital may have different meanings for women and men, as well as by job types, all analyses were stratified by sex and occupation. First, to compare proportions across the groups with and without suicidal ideation in the past year, we used the chi-square or Fisher’s exact test. Second, to identify associations between workplace social capital and suicidal ideation in the past year, we performed a series of binomial logistic regression analyses to calculate the unadjusted odds ratios (ORs) and 95% confidence intervals (CIs). Suicidal ideation in the past year was used as the dependent variable, and workplace social capital (reference = high quartile) was used as the explanatory variable. Model 1 included age group, and model 2 was additionally adjusted for marital status, educational attainment, and annual household income. Moreover, following a previous study [45], *p* values for linear trends were calculated by treating the four categories as ordinal variables. All statistical tests were two-tailed, and p values < 0.05 were regarded as indicating statistical significance. IBM SPSS for Windows (version 25.0; IBM Corp., Armonk, NY, USA) was used for all statistical analyses.

## Results

Of the 19,481 employees, 7255 completed the questionnaire (response rate: 37.2%). Participants who did not respond to the questions about suicidal ideation were excluded (*n* = 758), as were participants older than 65 years of age (*n* = 53), because 65 years is the normal retirement age in Japan. Participants who did not specify their occupation (*n* = 101) or educational attainment (*n* = 18) were also excluded. Finally, data from 6325 participants were analyzed, with a valid response rate of 32.5% (6325 of 19,481).

The age distributions (mean ± standard deviation [SD]) of the participants were 45.1 ± 10.6 years for men (*n* = 4030) and 42.5 ± 9.7 years for women (*n* = 2295). According to the quartile distribution, 25.9, 26.3, 28.1, and 19.7% of the participants were classified as having low, mid-low, mid-high, and high workplace social capital, respectively.

The participants’ characteristics and scores for workplace social capital by sex are shown in Table 1. Overall, the mean scores for workplace social capital (± SD) were 3.58 ± 0.81 for men and 3.59 ± 0.81 for women. The characteristics of those who showed higher workplace social capital can be summarized as follows: occupational status of researcher/academic, 20–29 years old, married, graduate school as final academic background, earning a high income, and no suicidal ideation in the past year. In total, 5.9% of men and 7.8% of women reporting experiencing suicidal ideation in the past year. Nearly half of the men described their occupation as researcher/academic, whereas more than half of the women described their occupation as clerk/administration. The largest age groups were 50–65 years for men and in the 40s for women. More than 60% of the men and 27.1% of the women had completed graduate school. More than 15% of the men and women had a household income of ≥ 12 million yen.

**Table 1:** Descriptions of participants’ characteristics and workplace social capital, Tsukuba, Japan (2017)

	Men				Women			
			Workplace social capital				Workplace social capital	
Characteristics	N	(%)	Mean	± SD	N	(%)	Mean	± SD
Overall	4030	(100)	3.58	± 0.81	2295	(100)	3.59	± 0.81
Suicidal ideation in the past year								
No	3794	(94.1)	3.61	± 0.79	2117	(92.2)	3.61	± 0.79
Yes	236	(5.9)	3.17	± 1.04	178	(7.8)	3.34	± 1.01
Occupation								
Researcher/academic	1975	(49.0)	3.59	± 0.80	704	(30.7)	3.65	± 0.84
Clerk/administration	992	(24.6)	3.57	± 0.80	1244	(54.2)	3.56	± 0.78
Technician/engineer	1063	(26.4)	3.58	± 0.83	347	(15.1)	3.58	± 0.85
Age group								
20–29	339	(8.4)	3.71	± 0.90	245	(10.7)	3.66	± 0.85
30–39	982	(24.4)	3.57	± 0.89	646	(28.1)	3.59	± 0.88
40–49	1150	(28.5)	3.56	± 0.83	818	(35.6)	3.59	± 0.78
50–65	1559	(38.7)	3.58	± 0.71	586	(25.5)	3.58	± 0.76
Marital status								
Married	3015	(74.8)	3.60	± 0.79	1492	(65.0)	3.64	± 0.76
Never married	913	(22.7)	3.56	± 0.87	666	(29.0)	3.52	± 0.89
Divorced/widowed	102	(2.5)	3.49	± 0.79	137	(6.0)	3.38	± 0.89
Educational attainment								
Graduate school	2456	(60.9)	3.63	± 0.79	621	(27.1)	3.62	± 0.83
University	857	(21.3)	3.54	± 0.86	850	(37.0)	3.58	± 0.83
College etc.	215	(5.3)	3.41	± 0.89	504	(22.0)	3.62	± 0.75
High school	502	(12.5)	3.54	± 0.74	320	(13.9)	3.53	± 0.82
Annual household income, JPY								
12 million or more	635	(15.8)	3.73	± 0.70	356	(15.5)	3.69	± 0.76
8–12 million	1536	(38.1)	3.61	± 0.77	605	(26.4)	3.61	± 0.74
4–8 million	1446	(35.9)	3.51	± 0.86	782	(34.1)	3.58	± 0.82
4 million or less	413	(10.2)	3.56	± 0.92	552	(24.1)	3.53	± 0.91

The characteristics of the men, classified by suicidal ideation in the past year, are summarized in Table 2. For all occupations, suicidal ideation in the past year was significantly associated with workplace social capital, and age group. Men with mid-to-high workplace social capital in the job groups of researcher/academic and technician/engineer were the least likely to report suicidal ideation, while those with high workplace social capital in the job group of clerk/administration were the least likely to report suicidal ideation. In all groups, men with low social capital were the most likely to report suicidal ideation.

**Table 2:** Comparisons of characteristics by suicidal ideation stratified by occupation (men), Tsukuba, Japan (2017)

		Overall, *n* = 4030			Researcher/academic, *n* = 1975			Clerk/administration, *n* = 992			Technician/engineer, *n* = 1063		
	Suicidal ideation	Yes	No	*P* value	Yes	No	*P* value	Yes	No	*P* value	Yes	No	*P* value
Variables		*N* = 236 (5.9)	*N* = 3794 (94.1)		*N* = 108 (5.5)	*N* = 1867 (94.5)		*N* = 69 (7.0)	*N* = 923 (93.0)		*N* = 59 (5.6)	*N* = 1004 (94.4)	
Workplace Social Capital				< 0.001			0.002			0.005			< 0.001
High		33 (4.3)	728 (95.7)		17 (4.4)	369 (95.6)		7 (4.1)	163 (95.9)		9 (4.4)	196 (95.6)	
Mid-high		41 (3.4)	1158 (96.6)		23 (3.9)	573 (96.1)		12 (4.4)	262 (95.6)		6 (1.8)	323 (98.2)	
Mid-low		53 (5.2)	971 (94.8)		24 (4.9)	467 (95.1)		20 (7.0)	264 (93.0)		9 (3.6)	240 (96.4)	
Low		109 (10.4)	937 (89.6)		44 (8.8)	458 (91.2)		30 (11.4)	234 (88.6)		35 (12.5)	245 (87.5)	
Age group				< 0.001			< 0.001			0.001			0.005
20–29		37 (10.9)	302 (89.1)		14 (11.5)	108 (88.5)		15 (12.4)	106 (87.6)		8 (8.3)	88 (91.7)	
30–39		77 (7.8)	905 (92.2)		43 (7.2)	551 (92.8)		16 (9.0)	162 (91.0)		18 (8.6)	192 (91.4)	
40–49		72 (6.3)	1078 (93.7)		28 (4.8)	552 (95.2)		23 (8.9)	234 (91.1)		21 (6.7)	292 (93.3)	
50–65		50 (3.2)	1509 (96.8)		23 (3.4)	656 (96.6)		15 (3.4)	421 (96.6)		12 (2.7)	432 (97.3)	
Marital status				< 0.001			< 0.001			0.127			0.001
Married		132 (4.4)	2883 (95.6)		57 (3.8)	1461 (96.2)		42 (6.0)	657 (94.0)		33 (4.1)	765 (95.9)	
Never married		96 (10.5)	817 (89.5)		47 (11.0)	379 (89.0)		25 (9.7)	232 (90.3)		24 (10.4)	206 (89.6)	
Divorced/widowed		8 (7.8)	94 (92.2)		4 (12.9)	27 (87.1)		2 (5.6)	34 (94.4)		2 (5.7)	33 (94.3)	
Educational attainment				0.108			0.595^a^			0.104			0.036
Graduate school		126 (5.1)	2330 (94.9)		98 (5.7)	1621 (94.3)		5 (3.3)	148 (96.7)		23 (3.9)	561 (96.1)	
University		61 (7.1)	796 (92.9)		7 (3.6)	189 (96.4)		36 (8.6)	381 (91.4)		18 (7.4)	226 (92.6)	
College etc.		15 (7.0)	200 (93.0)		2 (6.7)	28 (93.3)		9 (8.9)	92 (91.1)		4 (4.8)	80 (95.2)	
High school		34 (6.8)	468 (93.2)		1 (3.3)	29 (96.7)		19 (5.9)	302 (94.1)		14 (9.3)	137 (90.7)	
Annual household income, JPY				< 0.001			< 0.001			0.203			0.004
12 million or more		22 (3.5)	613 (96.5)		13 (3.5)	359 (96.5)		6 (5.4)	105 (94.6)		3 (2.0)	149 (98.0)	
8–12 million		67 (4.4)	1469 (95.6)		35 (4.1)	823 (95.9)		18 (5.8)	290 (94.2)		14 (3.8)	356 (96.2)	
4–8 million		99 (6.8)	1347 (93.2)		41 (6.8)	560 (93.2)		31 (6.9)	418 (93.1)		27 (6.8)	369 (93.2)	
4 million or less		48 (11.6)	365 (88.4)		19 (13.2)	125 (86.8)		14 (11.3)	110 (88.7)		15 (10.3)	130 (89.7)	

Statistical analyses were conducted with Chi-squared test

Percentage were shown in parenthesis

^a^Fisher’s exact test

The characteristics of the women, classified by suicidal ideation in the past year, are summarized in Table 3. Workplace social capital was significantly associated with suicidal ideation in the job groups of researcher/academic (*p* = 0.003) and clerk/administration (*p* = 0.023), but not technician/engineer (*p* = 0.053). Women with mid-to-low workplace social capital in the job groups of researcher/academic and technician/engineer were the least likely to report suicidal ideation, while those with mid-to-high workplace social capital in the job group of clerk/administration were the least likely to report suicidal ideation. In all groups, women with low social capital were the most likely to report suicidal ideation.

**Table 3:** Comparisons of characteristics by suicidal ideation stratified by occupation (women), Tsukuba, Japan (2017)

		Overall, *n* = 2295			Researcher/academic, *n* = 704			Clerk/administration, *n* = 1244			Technician/engineer, *n* = 347		
	Suicidal ideation	Yes	No	*P* value	Yes	No	*P* value	Yes	No	*P* value	Yes	No	*P* value
Variables		*N* = 178 (7.8%)	*N* = 2117 (92.2)		*N* = 46 (6.5)	*N* = 658 (93.5)		*N* = 106 (8.5)	*N* = 1138 (91.5)		*N* = 26 (7.5)	*N* = 321 (92.5)	
Workplace Social Capital				< 0.001			0.003			0.023			0.053
High		36 (7.4)	450 (92.6)		13 (7.6)	158 (92.4)		16 (6.9)	217 (93.1)		7 (8.5)	75 (91.5)	
Mid-high		34 (5.9)	545 (94.1)		10 (5.1)	187 (94.9)		19 (6.4)	276 (93.6)		5 (5.7)	82 (94.3)	
Mid-low		35 (5.5)	601 (94.5)		3 (1.9)	159 (98.1)		30 (7.7)	359 (92.3)		2 (2.4)	83 (97.6)	
Low		73 (12.3)	521 (87.7)		20 (11.5)	154 (88.5)		41 (12.5)	286 (87.5)		12 (12.9)	81 (87.1)	
Age group				< 0.001			0.421			< 0.001			0.001
20–29		32 (13.1)	213 (86.9)		6 (10.3)	52 (89.7)		24 (15.6)	130 (84.4)		2 (6.1)	31 (93.9)	
30–39		73 (11.3)	573 (88.7)		18 (7.2)	232 (92.8)		40 (13.2)	264 (86.8)		15 (16.3)	77 (83.7)	
40–49		51 (6.2)	767 (93.8)		12 (4.8)	238 (95.2)		31 (6.9)	416 (93.1)		8 (6.6)	113 (93.4)	
50–65		22 (3.8)	564 (96.2)		10 (6.8)	136 (93.2)		11 (3.2)	328 (96.8)		1 (1.0)	100 (99.0)	
Marital status				< 0.001			0.693			< 0.001			0.141
Married		83 (5.6)	1409 (94.4)		28 (6.0)	439 (94.0)		42 (5.3)	749 (94.7)		13 (5.6)	221 (94.4)	
Never married		78 (11.7)	588 (88.3)		16 (7.8)	190 (92.2)		51 (13.9)	315 (86.1)		11 (11.7)	83 (88.3)	
Divorced/widowed		17 (12.4)	120 (87.6)		2 (6.5)	29 (93.5)		13 (14.9)	74 (85.1)		2 (10.5)	17 (89.5)	
Educational attainment				0.174			0.734			0.006			0.802^a^
Graduate school		50 (8.1)	571 (91.9)		29 (7.1)	377 (92.9)		10 (11.6)	76 (88.4)		11 (8.5)	118 (91.5)	
University		77 (9.1)	773 (90.9)		8 (4.7)	163 (95.3)		61 (11.2)	483 (88.8)		8 (5.9)	127 (94.1)	
College etc.		32 (6.3)	472 (93.7)		6 (7.0)	80 (93.0)		21 (5.8)	344 (94.2)		5 (9.4)	48 (90.6)	
High school		19 (5.9)	301 (94.1)		3 (7.3)	38 (92.7)		14 (5.6)	235 (94.4)		2 (6.7)	28 (93.3)	
Annual household income, JPY				0.019			0.346			0.043			0.793
12 million or more		18 (5.1)	338 (94.9)		7 (4.1)	162 (95.9)		6 (4.5)	128 (95.5)		5 (9.4)	48 (90.6)	
8–12 million		37 (6.1)	568 (93.9)		12 (6.3)	177 (93.7)		20 (6.2)	304 (93.8)		5 (5.4)	87 (94.6)	
4–8 million		71 (9.1)	711 (90.9)		19 (8.7)	199 (91.3)		43 (9.7)	399 (90.3)		9 (7.4)	113 (92.6)	
4 million or less		52 (9.4)	500 (90.6)		8 (6.3)	120 (93.8)		37 (10.8)	307 (89.2)		7 (8.8)	73 (91.3)	

Statistical analyses were conducted with Chi-squared test

Percentage were shown in parenthesis

^a^ Fisher’s exact test

The results of a series of binomial logistic regressions for men are shown in Table 4. Overall, a significant association was observed between suicidal ideation in the past year and low social capital in the crude model (OR = 2.57, 95% CI = 1.72–3.83), model 1 (OR = 2.89, 95% CI = 1.93–4.33), and model 2 (OR = 2.80, 95% CI = 1.85–4.22). In all occupational groups, similar patterns were observed in statistical significance. *P* values for linear trends were statistically significant both overall and in the all job groups.

**Table 4:** Odds ratios for suicidal ideation in the past year associated with workplace social capital in men, Tsukuba, Japan (2017)

	Crude model			Model 1^a^			Model 2^b^		
Variables	OR	95% CI	*P* for liner trend	OR	95% CI	*P* for liner trend	OR	95% CI	*P* for liner trend
Overall (*n* = 4030)									
Workplace Social Capital (vs. High)			< 0.001			< 0.001			< 0.001
Mid-high	0.78	0.49–1.25		0.92	0.57–1.47		0.91	0.57–1.47	
Mid-low	1.20	0.77–1.88		1.44	0.92–2.25		1.43	0.91–2.25	
Low	2.57	1.72–3.83		2.89	1.93–4.33		2.80	1.85–4.22	
Researcher/academic (*n* = 1975)									
Workplace Social Capital (vs. High)			0.002			< 0.001			0.001
Mid-high	0.87	0.46–1.65		1.04	0.54–1.98		1.01	0.53–1.95	
Mid-low	1.12	0.59–2.11		1.38	0.72–2.63		1.41	0.73–2.72	
Low	2.09	1.17–3.71		2.43	1.35–4.35		2.41	1.33–4.36	
Clerk/administration (*n* = 992)									
Workplace Social Capital (vs. High)			0.001			0.001			0.001
Mid-high	1.07	0.41–2.76		1.26	0.48–3.30		1.28	0.49–3.36	
Mid-low	1.76	0.73–4.26		2.06	0.84–5.02		2.05	0.83–5.05	
Low	2.99	1.28–6.96		3.20	1.36–7.50		3.20	1.35–7.60	
Technician/engineer (*n* = 1063)									
Workplace Social Capital (vs. High)			< 0.001			< 0.001			< 0.001
Mid-high	0.41	0.14–1.15		0.47	0.16–1.34		0.46	0.16–1.32	
Mid-low	0.82	0.32–2.10		0.95	0.37–2.45		0.89	0.34–2.33	
Low	3.11	1.46–6.63		3.51	1.63–7.54		3.16	1.45–6.90	

Statistical analyses were conducted with binomial logistic regression

^a^ Adjusted for age group

^b^ Adjusted for age group, marital status, educational attainment, and annual household income. Overall results were also adjusted by occupation

The results for women were not uniform compared to men (Table 5). Significant associations were observed for overall samples, i.e., ORs for suicidal ideation was 1.77 even after adjusting possible confounders (Model 2) and liner trend for social capital categories were also clear in all models. In the job group of clerk/administration, similar significant associations and liner trends were observed between suicidal ideation and low social capital in the crude model (OR = 1.94, 95% CI = 1.06–3.56), model 1 (OR = 2.11, 95% CI = 1.14–3.90), and model 2 (OR = 1.97, 95% CI = 1.06–3.67). However, the patterns were different from occupations. In the job group of researcher/academic and technician/engineer, there were still elevated OR for low social capital (OR = 1.59 for researcher/academic and OR = 1.70 for technician/engineer, respectively) even thought it was not statistically significant, and in addition there were no clear dose response relationships due to low ORs for especially in mid-low social capital categories (e.g., OR = 0.24, 95%CI: 0.07–0.86 for researcher/academic in Model 2).

**Table 5:** Odds ratios for suicidal ideation in the past year associated with workplace social capital in women, Tsukuba, Japan (2017)

	Crude model			Model 1^a^			Model 2^b^		
Variables	OR	95% CI	*P* for liner trend	OR	95% CI	*P* for liner trend	OR	95% CI	*P* for liner trend
Overall (*n* = 2295)									
Workplace Social Capital (vs. High)			0.003			0.002			0.006
Mid-high	0.78	0.48–1.27		0.88	0.54–1.43		0.85	0.52–1.40	
Mid-low	0.73	0.45–1.18		0.83	0.51–1.34		0.76	0.47–1.24	
Low	1.75	1.15–2.66		1.92	1.25–2.93		1.77	1.15–2.71	
Researcher/academic (*n* = 704)									
Workplace Social Capital (vs. High)			0.282			0.232			0.295
Mid-high	0.65	0.28–1.52		0.70	0.30–1.65		0.69	0.29–1.65	
Mid-low	0.23	0.06–0.82		0.25	0.07–0.89		0.24	0.07–0.86	
Low	1.58	0.76–3.28		1.67	0.80–3.49		1.59	0.75–3.37	
Clerk/administration (*n* = 1244)									
Workplace Social Capital (vs. High)			0.010			0.008			0.017
Mid-high	0.93	0.47–1.86		1.04	0.52–2.08		1.00	0.49–2.03	
Mid-low	1.13	0.60–2.13		1.28	0.68–2.42		1.22	0.64–2.34	
Low	1.94	1.06–3.56		2.11	1.14–3.90		1.97	1.06–3.67	
Technician/engineer (*n* = 347)									
Workplace Social Capital (vs. High)			0.380			0.484			0.534
Mid-high	0.65	0.20–2.15		0.72	0.22–2.39		0.71	0.21–2.42	
Mid-low	0.26	0.05–1.28		0.30	0.06–1.49		0.28	0.05–1.47	
Low	1.59	0.59–4.25		1.68	0.62–4.56		1.70	0.60–4.76	

Statistical analyses were conducted with binomial logistic regression

^a^ Adjusted for age group

^b^ Adjusted for age group, marital status, educational attainment, and annual household income. Overall results were also adjusted by occupation

## Discussion

This study aimed to evaluate the association between workplace social capital and suicidal ideation in the past year among workers in Japan. We found that workplace social capital was inversely associated with suicidal ideation. Stratified analysis showed that the low workplace social capital was associated with high suicidal ideation compared with workers with high social capital in both sexes, and in all type of job groups. Our findings were in line with previous studies which reported in a residential or geographical area. For example, in a cross-sectional study involving 10,094 older residents in Japan, mistrust and a lack of reciprocity at the individual level were associated with suicidal ideation even after controlling for psychological distress [19]. In another study among Japanese residents, individual-level social trust reduced the probability of suicidal ideation [23]. Precedent studies of the relationship between social capital and suicide were mainly conducted in neighborhood/community settings. Therefore, our result advanced the existing literature with evidence in occupational settings.

The mechanisms underlying the association between perceived social capital at work and suicidal ideation might be similar to those in neighborhood/community settings. First, workplace social capital might buffer the effect of adverse work-related factors on mental health. Precedent studies have shown the associations between mental health and job stress or job insecurity were moderated by workplace social capital [28, 29]. Secondly, workplace social capital could enhance support from colleagues or supervisors, which could be considered as important health resource. Thirdly, more cohesive workplaces are likely to be more effective in maintaining healthy norms [13], e.g. encouraging co-workers who seems exhausted to meet industrial physician or to take paid holidays.

In the present study, the prevalence of suicidal ideation in the past year was 5.9% for men and 7.8% for women. These values were higher than those reported in a national survey using the same questionnaire items (5.0% for men and 5.3% for women, excluding non-responses) [9]. Although suicidal ideation did not directly reflect suicide commitment, the results are in line with those from previous studies in which overwork-related suicide was more common in the scientific research compared with other industries [8]. The discrepancy between our results and the national survey might reflect the differences of the age distribution of the participants because the national survey included a large number of respondents in their 60’s and 70’s; adults in this age range generally have less suicidal ideation compared with their younger counterparts.

Notably, there is a suggestion that female researchers/engineers in mid-to-low workplace social capital may be protected from suicide ideation. A key commonality exists between scientific communities involving researchers, and professional workers in Japan: they are male-dominated. Although the Japanese Equal Employment Opportunity Law was enacted in 1986, as of 2016, women still accounted for only 15.3% of all researchers and 24.8% of all full-time employees in Japan [46, 47]. In the present study, one-fourth of the participants in the job groups of researchers/academic and technician/engineer were women, which is slightly higher than the national average. However, men still comprise the overwhelming majority in their job groups. It is plausible that female researchers and professional workers cannot make use of workplace social capital to maintain their health in a male-dominated workplace. Another possible explanation for the association found between low suicidal ideation and mid-to-low workplace social capital is that women are more susceptible than men to factors outside the workplace. Another study found that a high level of neighborhood social capital affected women more than men [48]. It is possible that women with mid-to-low workplace social capital are compensated by a high level of neighborhood social capital, which reduces suicidal ideation. However, it should be noted that the sample size of female researchers with suicidal ideation was relatively small in the present study, which may be lead to biased estimation of the result. Therefore, future studies should be conducted with an adequate sample size of female researchers as well as the interaction between workplace and community social capital.

### Strengths and limitations

The present study had several strengths. To our knowledge, this is the first study to demonstrate clearly the association between workplace social capital and suicidal ideation in Japan. Previous studies on the relationship between social capital and suicide have been conducted in geographically-defined residential areas. In addition, our study had a considerable sample size, with a total of 6325 participants, including a large number of scientific researchers. Few studies to date on workplace social capital have included researchers as their participants. Furthermore, our results were stratified by sex and occupation, after adjusting for socioeconomic status, including household income. Most previous studies have failed to adjust for income, even if adjusting for other indicators of socioeconomic status (e.g., education and occupation).

However, the present study also had some limitations. First, because of the cross-sectional design of our study, causal relationships cannot be made. It is possible that employees with suicidal ideation may have been less likely to build trust, solidarity, and reciprocity in the workplace. As a result, they might report lower level of social capital. A longitudinal study is needed to disentangle the causal relationship between workplace social capital and suicidal ideation. Second, our survey was conducted by web-based method which could generally induce low response rate compared to other methods (e.g., paper-based survey). However, researchers and related occupations who are familiar with many types of surveys accounted for the majority of our target population. Therefore, we could consider the difference to response rate between web-based and paper-based methods was ignorable in our target. Regardless of the survey methods, we still need to caution about selection bias. Because the experience of suicidal ideation is sensitive personal information, a number of participants, especially those with suicidal ideation might have been reluctant to respond to the questionnaire. In that case, selection was only related to outcome measure, then it might affect reliability of our study. If the selection was related both exposure and outcome simultaneously, i.e., subject who evaluated their workplace social capital was low and thought about suicide were more reluctant to participate, then our effect estimates might be overestimated. Third, our measures on both exposure and outcome relied on subjective measures, which may introduce common-method bias, including under- or over-reporting, and recall issues. However, surveys on suicidal ideation are generally assessed by self-report, and could not avoid those issues. Forth, our results showed the association between suicidal ideation “in the past year” and “current” workplace social capital. It might seem to be straightforwardly suffered from reverse causation by the temporal relationship between exposure and outcome. However, because social capital is fostered over long time [13], we could consider the level of workplace social capital is quite similar levels even if we asked the status “in the past year”. Therefore, we can assume that time difference has not much impact on our results. Fifth, because of the nature of the study design, we did not have group level identifier; therefore, we could not conduct a multi-level analysis. However, in addition to contextual assessment, individual assessment of social capital has been considered an important determinant of individual health [38, 49]. Sixth, our target population was not a random sample from the general working population in Japan. Some caution is required when generalizing the findings to workers in general. Seventh, not all factors related to suicide was considered in the current study. Other work-related factors, including job stress [50], and objective work-related indicators such as working hours, length of overtime work, or paid holiday utilization rates, should also be considered in future study. Lastly, although health outcomes might have been affected by neighborhood social capital, we did not assess social capital outside the workplace in the present study.

## Conclusion

Although future studies are needed to explore the effectiveness of workplace social capital for suicidal prevention, we concluded that individual workplace social capital is protective against suicidal ideation in the past year among Japanese workers. The findings of the current study highlight the role of workplace social capital and demonstrated implications to occupational health strategy. Developing interventions to promote workplace social capital might lead to achievement of suicide prevention.

## Data Availability

The data that support the findings of this study are available from TSCN but restrictions apply to the availability of these data, which were used under license for the current study, and so are not publicly available. Data are however available from the authors upon reasonable request and with permission of TSCN.

## Abbreviations

CI: Confidence interval
GHQ: General Health Questionnaire
OR: Odds ratio
SD: Standard deviation
TSCN: Tsukuba Science City Network
WHO-CIDI: WHO Composite International Diagnostic Interview
